# Implementing a trilineage differentiation in the ReproTracker assay for improved teratogenicity assessment

**DOI:** 10.3389/ftox.2025.1645842

**Published:** 2025-09-25

**Authors:** José M. Horcas-Nieto, Sabine Hartvelt, Luke Flatt, Jing Fang, Esther Lam, Gaonan Zhang, Romy Van Vliet, Marleen Feliksik, Tom Zwetsloot, Connor Philippo, Giel Hendriks, Amer Jamalpoor

**Affiliations:** Toxys B.V., Oegstgeest, Netherlands

**Keywords:** developmental toxicity, new approach methodologies (NAM), ReproTracker, human iPSCs, ICHS5 validation

## Abstract

**Introduction:**

Exposure to teratogenic compounds during pregnancy can lead to significant birth defects. Given the considerable variation in drug responses across species, along with the financial and ethical challenges associated with animal testing, the development of advanced human-based in vitro assays is imperative for effectively identifying potential human teratogens. Previously, we developed a human induced pluripotent stem cells (hiPSCs)-based biomarker assay, ReproTracker, that follows the differentiation of hiPSCs into hepatocytes and cardiomyocytes. The assay combines morphological profiling with the assessment of time-dependent expression patterns of cell-specific biomarkers to detect developmental toxicity responses.

**Methods:**

To further increase the predictability of the assay in identifying potential teratogens, we added differentiation of hiPSCs towards neural rosette-like cells. We evaluated the performance of the extended assay with a set of 51 well-known in vivo teratogens and non-teratogens, including the compounds listed in the ICH S5 reference list.

**Results:**

The optimized assay correctly identified (neuro)developmental toxicants that were not detected in the hepatocyte and cardiomyocyte differentiation assays. These compounds selectively downregulated gene and protein expression of the neuroectodermal marker PAX6 and/or neural rosette marker NESTIN in a concentration-dependent manner and disrupted the differentiation of hiPSCs towards neural rosette-like cells. Overall, based on the current dataset, the addition of neural commitment improved the assay accuracy (from 72.55% to 86.27%) and sensitivity (from 67.50% to 87.50%), when compared to the previously described assay.

**Discussion:**

In summary, trilineage differentiation expanded the spectrum of teratogenic agents detectable by ReproTracker, making the assay an invaluable tool for early in vitro teratogenicity screening.

## 1 Introduction

Exposure to teratogenic compounds during pregnancy can result in serious birth defects. Epidemiological studies indicate that, every year, millions of newborns suffer from adverse birth outcomes (Chawanpaiboon et al., 2019; Blencowe et al., 2019), often due to prenatal exposure to teratogenic chemicals (Environmental Protection Agency, 2013; Voit et al., 2022; Louisse et al., 2012). Such compound exposures can lead to embryonic lethality, growth retardation, low birth weight, or severe congenital disorders (Frontiers et al., 2000). Currently, *in vivo* approaches using laboratory animals are still considered the gold standard for developmental and reproductive toxicity (DART) testing. However, such methods are resource-intensive, time-consuming, ethically debatable, and have the inherent drawbacks of interspecies variations. A recent study found a 45% discrepancy between outcomes in rats and rabbits, the two most common species used for DART testing (Roos et al., 2024), further highlighting the complications of animal testing and the need for advanced human *in vitro* assays.

Over the past decades, several assays, such as TeraTox, the human pluripotent stem cell test, and the DevTox Germ Layer Reporter Platform, were developed using directed differentiation of human pluripotent or embryonic stem cells (hiPSCs/hESCs) to assess the effect of chemical exposure on expression of the early germ-layer genes as a surrogate for teratogenicity (interchangeably referred to as developmental toxicity) assessment (Jaklin et al., 2020; Gamble et al., 2022). Alternatively, other assays like the DevTox quickPredict assay applied metabolomics on undifferentiated hiPSCs/hESCs to predict developmental toxicity (Palmer et al., 2013). These assays are generally fast (2–7 days) and can be applied for medium throughput screening. However, focusing solely on the effects of teratogenic compounds during early germ layer differentiation may overlook critical biological processes in later developmental stages, which could be highly susceptible to teratogens. Consequently, it remains unclear whether such assays can accurately capture the key biological pathways relevant to early embryonic development.

Previously, we developed ReproTracker, a more advanced hiPSCs-based biomarker assay to predict the developmental toxicity potential of new drugs and chemicals with high sensitivity and specificity (Jamalpoor, 2022). The ReproTracker assay follows the differentiation of hiPSCs into two crucial stages for early embryo-development: meso- and endodermal commitment, leading to the development of cardiomyocytes and hepatocytes, respectively. The assay combines morphological profiling with the assessment of temporal expression patterns of cell-specific biomarkers to detect developmental toxicity responses. However, the assessment of compounds inducing neurodevelopmental toxicity was not captured in the assay. These chemicals have become highly relevant as the number of drugs inducing neurodevelopmental abnormality is quickly rising (Grandjean and Landrigan, 2006; Tran and Miyake, 2017). Therefore, to increase the biological coverage and predictability of ReproTracker in identifying human teratogens, we optimized the assay by inclusion of the ectodermal germ layer, from which neural cells can be developed.

HiPSCs have been previously shown potential to differentiate towards neural progenitor cells (NPCs) (Kang et al., 2017). NPCs have been widely characterized by the expression of several markers such as *PAX6, NGN2*, *SOX2 *and *SOX1* (Kang et al., 2017; Lee et al., 2016). These cells can be further differentiated into neural rosettes, which resemble the early development of the neural tube *in vivo* (Zhang et al., 2001; Dreser et al., 2020; Wilson, 2006). Neural rosettes are *in vitro* structures, containing elongated neuroepithelial cells which arrange spatially in a blossom-like fashion (Miotto et al., 2023). Interference of the differentiation process of hiPSCs into neural rosettes has been investigated and used as a quantifiable endpoint for *in vitro* neurodevelopmental toxicity testing (Dreser, 2020).

In the present study, we introduce the differentiation of hiPSCs towards neural rosette-like cells in the ReproTracker assay and its potential to detect neurodevelopmental toxicity *in vitro*. Moreover, the applicability of ReproTracker with the new trilineage differentiation (i.e., cardiomyocytes, hepatocytes, neural rosette-like cells) was evaluated by testing 51 well-known human and animal teratogenic and non-teratogenic compounds. Obtained data shows that the inclusion of neural rosette differentiation improves ReproTracker’s performance, resulting in an overall accuracy of 86.27% with a sensitivity and a specificity of 87.50% and 81.82%, respectively. Overall, the trilineage differentiation in ReproTracker offers broader biological coverage and has the potential to improve teratogenicity prediction without the use of animal testing.

## 2 Materials and methods

### 2.1 Chemicals

Chemicals used in this study were purchased from Sigma-Aldrich (Schelldorf, Germany) or Selleck Chemicals (Berlin, Germany). All tested chemicals and their CAS numbers are listed in Table 1.

**TABLE 1:** Compounds screened in the ReproTracker assay.

#	Compounds	*CAS No.*	In vivo classification			Human therapeutic cmax (µM)	ReproTracker results					References
			R	RB	H		LOAEL (µM)	Cardiomyocyte- like cells	Hepatocyte-like cells	Neural rosette-like cells	Final classification	
1	Acitretin*	55079-83-9	T	T	T	0.03–0.9	≤0.2	Positive	Positive	Positive	Positive	R3 I (2021); Schulz et al. (2020)
2	Aspirin*	50-78-2	T	N.d	T	111–1110	500	Negative	Positive	Negative	Positive	R3 I (2021); Schulz et al. (2020)
3	Bosentan*	147536-97-8	T	N.d	N.d	1.3–2.9	15.6	Positive	Equivocal	Negative	Positive	R3 I (2021); Schulz et al. (2020)
4	Busulfan*	55-98-1	T	T	T	0.2	0.5	Negative	Negative	Positive	Positive	R3 I (2021); Schulz et al. (2020)
5	Carbamazepine*	298-46-4	T	T	T	8.5–33.9	≤7.8	Positive	Positive	Positive	Positive	R3 I (2021); Schulz et al. (2020)
6	Cisplatin*	15663-27-1	T	N.d	N.d	14.3	0.0016–0.0065	Positive	Negative	Positive	Positive	R3 I (2021); Schulz et al. (2020), Hassan et al. (2016)
7	Clarithromycin	81103-11-9	T	T	N.d	0.2–1.5	12.5	Negative	Negative	Positive	Positive	R3 I (2021); Schulz et al. (2020), Andersen et al. (2013), GM et al. (2015)
8	Cyclophosphamide*	6055-19-2	T	T	T	35.8–89.6	37.5	Negative	Negative	Positive	Positive	R3 I (2021); Schulz et al. (2020)
9	Cytarabine*	147-94-4	T	N.d	T	0.2–2.1	Positive	Positive	Negative	Positive	Positive	R3 I (2021); Schulz et al. (2020)
10	Dabrafenib*	1195765-45-7	T	N.d	N.d	N.a	0.0125	Negative	Negative	Positive	Positive	R3 I (2021); Schulz et al. (2020)
11	Dasatinib*	302962-49-8	T	N.d	N.d	0.07	0.00003–0.00013	Positive	Negative	Positive	Positive	R3 I (2021); He et al. (2021)
12	Dexamethasone	50-02-2	T	T	T	0.1–0.7	1.95	Negative	Negative	Positive	Positive	R3 I (2021); Schulz et al. (2020), FSC of Japan (2018), Cheng et al. (2014), Walker (1967)
13	Diltiazem^a^ ^,^ ^b^	33286-22-5	T	T	N.d	0.06–0.3	≤7.8	Positive	Positive	Positive	Positive	R3 I (2021); Schulz et al. (2020), Ariyuki (1975)
14	Fingolimod^a^ ^,^ ^b^	162359-56-0	T	N.d	T	0.003–0.05	≤0.25	Positive	Positive	Negative	Positive	Schulz et al. (2020), Teixidó et al. (2018), Yavuz et al. (2017)
15	Fluconazole*	86386-73-4	T	T	T	6.5–19.6	125	Negative	Negative	Positive	Positive	R3 I (2021); Schulz et al. (2020)
16	5-Fluorouracil*	51-21-8	T	T	T	0.4–2.3	N.d. up to 1.3	Negative	Negative	Negative	Negative**	R3 I (2021); Schulz et al. (2020)
17	Flusilazole	51-21-8	T	T	T	N.a	7.8–15.6	Positive	Positive	Positive	Positive	Aschner et al. (2017)
18	Hydroxyurea*^a^	127-07-1	T	T	T	263	≤2.0	Negative	Positive	Positive	Positive	R3 I (2021); EMA (2019)
19	Ibrutinib*	936563-96-1	T	T	N.d	0.1	0.8	Negative	Positive	Negative	Positive	R3 I (2021); Ohtani et al. (2022)
20	Ibuprofen*	15687-27-1	T	N.d	T	72.7–145.4	≤31.3	Positive	Positive	Positive	Positive	R3 I (2021); Schulz et al. (2020)
21	Imatinib*^a^	152459-95-5	T	N.d	N.d	1.5	0.1	Negative	Positive	Positive	Positive	R3 I (2021); Schulz et al. (2020), Hensley and Ford (2003)
22	Isotretinoin*	4759-48-2	T	T	T	0.004–0.008	≤0.0041	Positive	Positive	Positive	Positive	R3 I (2021); Schulz et al. (2020)
23	Lenalidomide	191732-72-6	T	NT	N.d	0.2–3.9	≤0.06	Positive	Positive	Negative	Positive	R3 I (2021); Schulz et al. (2020)
24	Methimazole^a^ ^,^ ^b^	60-56-0	T	NT	T	4.4–20.2	250	Positive	Negative	Positive	Positive	Schulz et al. (2020), Lee et al. (2019)
25	Methanol	67-56-1	T	NT	NT (presumed)	98.7	N.d. up to 1000	Negative	Negative	Negative	Negative	R3 I (2021); Schulz et al. (2020)
26	Methotrexate*	59-05-2	T	T	T	0.02–1.1	N.d. up to 0.122	Negative	Negative	Negative	Negative**	R3 I, 2021; Schulz et al. (2020)
27	Methylmercury^a^ ^,^ ^b^	115-09-3	T	N.d	N.d	0.001–0.03	N.d. up to 0.31	Negative	Negative	Negative	Negative**	R3 I (2021); Schulz et al. (2020), ECHA, 2024; Brown et al. (2012)
28	Mirex	2385-85-5	T	N.d	T	N.a	0.0049	Negative	Negative	Positive	Positive	R3 I (2021); Schulz et al. (2020), Faroon and Wohlers (2020)
29	Pazopanib*^a^	444731-52-6	T	T	n.d	91.4–251.4	0.5	Negative	Positive	Negative	Positive	R3 I (2021); EMA (2023)
30	Phenytoin*	57-41-0	T	T	T	39.6–79.3	31.3	Negative	Positive	Positive	Positive	R3 I (2021); Schulz et al. (2020)
31	Pomalidomide*	19171-19-8	T	T	T (presumed)	0.3–0.4	≤12.5	Positive	Positive	Positive	Positive	R3 I (2021); Schulz et al. (2020)
32	Ribavirin*	36791-04-5	T	T	N.d	3.2	≤1	Negative	Negative	Positive	Positive	R3 I (2021); Glue et al. (2000)
33	Tacrolimus*^a^	109581-93-3	T	T	N.d	0.0006–0.03	≤0.2	Equivocal	Positive	Positive	Positive	R3 I (2021); Schulz et al. (2020)
34	Thalidomide*^a^	50-35-1	T	T	T	0.2–31	≤0.08	Positive	Positive	Negative	Positive	R3 I (2021); Schulz et al. (2020)
35	Topiramate*	97240-79-4	T	T	T	5.9–29.5	125	Negative	Negative	Positive	Positive	R3 I (2021); Schulz et al. (2020)
36	Tretinoin (Retinoic acid)*	302-79-4	T	T	T	1.3	≤0.3	Positive	Positive	Positive	Positive	R3 I (2021); Schulz et al. (2020)
37	Trimethadione*	127-48-0	T	N.d	T	139.7–279.4	N.d. up to 1000	Negative	Negative	Negative	Negative	R3 I (2021); Schulz et al. (2020)
38	Valproic acid*^a^	99-66-1	T	T	T	1421	250	Negative	Positive	Negative	Positive	R3 I (2021); Schulz et al. (2020)
39	Vismodegib*	879085-55-9	T	N.d	T (presumed)	5.9–7.6	≤1	Equivocal	Positive	Positive	Positive	R3 I (2021); Lear et al. (2023)
40	Warfarin^a^ ^,^ ^b^	81-81-2	T	N.d	T	3.2–9.7	62.5–250	Positive	Positive	Negative	Positive	Schulz et al. (2020), Howe and Webster (1990)
41	Amoxicillin^a^ ^,^ ^b^	26787-78-0	NT	N.d	N.d	1.4–41	N.d. up to 500	Negative	Negative	Negative	Negative	Schulz et al. (2020)
42	Cetirizine*^c^	83881-51-0	NT	NT	N.d	0.3–1.5	N.d. up to 125	Negative	Negative	Negative	Negative	R3 I (2021); Schulz et al. (2020)
43	Folic acid^a^ ^,^ ^b^	59-30-3	NT	NT	NT	0.002–0.01	N.d. up to 1.95	Negative	Negative	Negative	Negative	R3 I (2021); Schulz et al. (2020), Menegola et al. (2001), Brent (1964)
44	Hydrochlorothiazide^a^ ^,^ ^b^	58-93-5	NT	N.d	N.d	0.1–0.2	≤62.5	Positive	Positive	Negative	Positive	(R3 I, 2021; Therapeutic Goods Administration Australia, 2010)
45	Metoclopramide^a^ ^,^ ^b^	364-62-5	NT	NT	NT	0.03–0.5	N.d. up to 62.5	Negative	Negative	Negative	Negative	(R3 I, 2021; Schulz et al., 2020; Broussard and Richter, 1998)
46	1,2-propylene glycol^a^ ^,^ ^b^	57-55-6	NT	NT	NT	0.3–3.1	N.d. up to 1000	Negative	Negative	Negative	Negative	(R3 I, 2021; Schulz et al., 2020)
47	Saccharin^a^	82385-42-0	NT	NT	N.d		N.d. up to 250	Negative	Negative	Negative	Negative	
48	Saxagliptin*^c^	361442-04-8	NT	NT	N.d	0.03–0.08	N.d. up to 250	Negative	Negative	Negative	Negative	(R3 I, 2021; Schulz et al., 2020)
49	Sitagliptin^a^ ^,^ ^b^	654671-77-9	NT	NT	N.d	0.1–0.9	N.d. up to 1000	Negative	Negative	Negative	Negative	(R3 I, 2021; Schulz et al., 2020; Szabadfi et al., 2014; Beats and Suppression, 2005)
50	Thiamine^a^ ^,^ ^b^	67-03-8	NT	NT	NT	0.4	N.d. up to 1000	Negative	Negative	Negative	Negative	(R3 I, 2021; Schulz et al., 2020; ECHA, 2024)
51	Vildagliptin*^c^ ^,^ ^a^	274901-16-5	NT	NT	N.d	0.6–1	125	Negative	Negative	Positive	Positive	(R3 I, 2021; Schulz et al., 2020)

Abbreviations: R, rat; RB, rabbit; H, human; NT, non-teratogen; T, teratogen; n.d, not determined. * ICH S5 chemicals screened in the ReproTracker assay. **Compounds exhibited highly cytotoxicity profiles leading to top tested concentrations at ≤1 µM.

^a^ Dose-range finding data for this compound were previously published and not newly generated for the current study.

^b^ Cardiomyocyte and hepatocyte ReproTracker data for this compound were previously published (Jamalpoor et al., 2022) and not newly generated for the current study. Green color indicates non-teratogen/negative, red indicates teratogen/positive and orange color indicates equivocal outcomes.

^c^ Compound did not induce MEFL, in rat and rabbit at an exposure multiple (AUC, and Cmax) of >25 fold at the Maximum Human Recommended Dose and has therefore been recommended as negative control by the ICH S5 Guideline on detection of reproductive and developmental toxicity for human pharmaceuticals (step 5).

### 2.2 Human iPSC culture

Human induced pluripotent stem cells (hiPSCs) (cat no.: A18945; ThermoFisher Scientific) were cultured in mTeSR™1 (cat no.:85851; STEMCELL Technologies; Koln, German) supplemented with mTeSR™1 5X Supplement (cat no.:85852; STEMCELL Technologies) and 0.5% v/v Penicillin-Streptomycin (Gibco; Bleiswijk, Netherlands). Cells were cultured on Matrigel™ (Corning)-coated (80 μg/mL) cell culture Petri dishes (Nunc EasYDish; ThermoFisher Scientific). The medium was changed every day, and cells were split every 3–4 days at a confluency of approximately 80%. HiPSCs were maintained at 37 °C and 5% CO_2_. Mycoplasma test was performed routinely.

### 2.3 Differentiation of hiPSCs into trilineage differentiation

Prior to differentiation, the cells were harvested and dissociated into single cells using Gibco™ TrypLE™ Select (ThermoFisher Scientific; Bleiswijk, Netherlands) for 5 min at 37 °C. Cells were seeded in Matrigel™-coated 24-well plates (ThermoFisher Scientific) in mTeSR™1 containing RevitaCell™ Supplement (ThermoFisher Scientific). Differentiation towards cardiomyocytes and hepatocytes cells was performed as described previously (Jamalpoor, 2022). Neural rosette-like differentiation was carried out using two methods: a previously described protocol (Fedorova, 2019), referred to as the replating protocol, and an in-house optimized method, termed the one-step protocol, which eliminated the need for cell passaging at D7. Briefly, differentiation towards neural rosette-like cells was instigated 24 h after seeding (D0), using rosette differentiation base N2B27 medium, consisting of DMEM/F-12 supplemented with GlutaMAX™ Supplement (ThermoFisher Scientific), MEM Non-Essential Amino Acids Solution (Gibco), Penicillin-Streptomycin (Gibco), N-2 Supplement (100X) (ThermoFisher Scientific) and B-27™ Supplement (50X) minus vitamin A (ThermoFisher Scientific), RevitaCell™ Supplement (ThermoFisher Scientific) or CloneR™2 (STEMCELL Technologies), and the small molecule LDN 193189 dihydrochloride (Tocris). The cells were cultured in this medium until D3. From D3 until the end of the differentiation protocol (D13) (one-step protocol), cells were cultured in N2B27 medium without ROCK inhibitor or LDN193189. In the case of the replating protocol, cells were replated at D7 and grown in the same medium until day 13. At the end of the differentiation, morphology of the cells was assessed using an Operetta CLS (Revvity) instrument. The expected morphology for each of the lineages, to ensure data acceptance and correct differentiations, is further described in Section 2.9 of the methods section. The trilineage differentiation was replicated in 96-well plates (ThermoFisher Scientific and Revvity Phenoplate) using the same differentiation protocols with an adapted number of cells for the seeding. And termination at D14.

### 2.4 Concentration-range selection and cytotoxicity assessment

Cytotoxicity of each compound was assessed in hiPSCs using AlamarBlue cell viability assay (Sigma-Aldrich) using undifferentiated hiPSCs as previously described (Jamalpoor, 2022). In short, hiPSCs were exposed to 20 consecutive concentrations of the test compounds using 1 mM or the maximum soluble concentration as the top concentration. Test compounds were added to the medium prior to the refreshes at D0 and D3. Cytotoxicity was estimated at D7; upon completion of the experiments, cells were incubated with resazurin solution (0.01 mg/mL in PBS) and measured absorbance (excitation 530 nm and emission 590 nm). Results were normalized against vehicle controls (0.1% v/v DMSO).

### 2.5 RNA isolation, reverse transcription and quantitative real-time qPCR

Cells were harvested, and total RNA was isolated using the Maxwell^®^ RSC simply RNA Cells Kit (cat. no.: #AS1390; Promega) on a Maxwell automated purification instrument (Maxwell RSC 48; Promega). One-step Quantitative real-time PCR (qRT-PCR) was performed in a 384-well plate format using 10 ng of RNA and PrimeTime™ One-Step RT-qPCR Master Mix (cat. no.: #10007067; IDT) with the specific primer-probes (IDT) at a final concentration of 500 nM and 250 nM, respectively, on a QuantStudio 5 instrument (Applied Biosystems). Each individual primer was paired with its corresponding fluorophore and quencher and adjusted accordingly depending on gene abundance. Primer and probe sequences are described in Supplementary Table S1.

Relative gene expression levels were calculated using the 2^−ΔΔ^ Ct or logΔCT methods (Livak and Schmittgen, 2001), and normalized to expression of the reference gene, *GAPDH*. Data acceptance was based on the appropriate expression of the assessed biomarker genes in the differentiated populations, at appropriate times during differentiation. Failure to observe the expected temporal expression of key, lineage-specific biomarkers, during the cell fate choices associated with each differentiation protocol, would result in the experiment being invalidated. Biomarker acceptance criteria include ranges of Ct values (<35 Ct value) for both housekeeping genes and genes of interest, as well as limited variation between technical replicates (±0.5 standard deviation).

### 2.6 Immunostaining

For neural rosette-like cells’ imaging, cells were washed with PBS and subsequently, cells were fixed using 4% paraformaldehyde in PBS. Permeabilization was performed using 0.1% Triton-X in PBS for 10 min at room temperature. Cells were blocked using 10% donkey serum diluted in PBS +0.1% Tween20 for 10 more minutes. Incubation with primary antibody overnight at 4 °C was performed to stain the cells. The cells were washed 3 times with PBS +0.1% Tween20 and incubated with a secondary antibody in PBS for 1 h at room temperature. Cellular nuclei were stained using Hoechst H33342 (CAS# C875756-97-1, Sigma Aldrich in a 1:5000 dilution). Imaging was done using an Operetta CLS (Revvity) instrument. Primary and secondary antibodies’ concentrations are described in Supplementary Table S2.

To quantify PAX6-and NESTIN-positive cells, Hoechst-stained nuclei were selected based on their fluorescent signal above the background. Cells were also excluded if touching the edge of the fields of view. Also, size exclusion was applied to avoid clustered nuclei being counted as single objects. Fluorescence measurements of PAX6 were performed within the defined nuclei and intracellularly for NESTIN. Then, a minimal fluorescence threshold was defined, resulting in the selection of PAX6-positive cells and NESTIN-positive cells. Finally, the percentages of PAX6-and NESTIN-positive cells were calculated using Revvity Harmony™ Software.

### 2.7 Compound testing

Compound stock solutions were prepared in dimethyl sulfoxide (DMSO; Sigma-Aldrich), DPBS (Gibco), or ddH_2_O, depending on solubility. All compounds were checked for precipitation in vehicle and culture medium.

All compounds were tested at five consecutive concentrations in two-fold dilution steps. The top concentration was determined as the lowest concentration that induced a maximum of 20% cytotoxicity in the concentration range finding experiments. Compounds were then diluted 2-fold using serial dilutions. Compounds were added to the differentiation medium prior to the refreshes at D0, D3, D6 and D10 for the neural rosette-like commitment, at D0, D2, D6, and D10 for the cardiac differentiation and at D0, D3, D7, D10, D14 and D17 for the hepatocyte differentiation.

To follow morphological changes throughout the hepatocyte and neural differentiation protocol, brightfield images of the cells were acquired after each medium refresh using an iRiS digital cell imaging microscope (Logos Biosystems) or an Operetta CLS (Revvity) instrument. Images were scored based on visual assessment. Cardiac contractability of cardiomyocytes was evaluated visually using an iRiS digital cell imaging microscope (Logos Biosystems).

All chemicals were tested in the complete assay exclusively for the manuscript and no data was derived from previous studies unless clearly stated.

### 2.8 Teratogenicity prediction in the ReproTracker assay

In ReproTracker, teratogenicity prediction of test compounds is based on a Weight of Evidence (WoE) approach, which considers both the significant decrease in biomarker expression and the morphological/functional changes following exposure to test compounds throughout differentiation assays.

For a biomarker to be considered significantly downregulated upon compound exposure, a threshold is established. This threshold, for any given biomarker, is defined as the average minus one time the standard deviation (SD) of the biomarker expression levels in solvent-exposed control cultures throughout the experiment. Reduced expression of the selected biomarkers below the set threshold in a concentration-dependent manner, upon exposure to a minimum of 2 consecutive non-cytotoxic concentrations would be considered significant (noted as +). A decrease in expression below the threshold without a clear dose-response would lead to an equivocal classification (noted as (+)). An increase in expression or a lack of response would be considered negative (noted as -). Disruption of morphology/functionality of the cultures upon compound exposure at the two highest tested concentrations in all biological replicates is considered as a morphological disruption (noted as +). Absence of significant effects results in a negative score (noted as -).

For each differentiation assay, the expression of two biomarkers and a morphological/functional read-out are considered. In the hepatocyte and cardiomyocyte differentiation, *FOXA2* and *BMP4* are considered germ-layer-specific biomarkers, while *AFP* and *MYH6* are considered cell-specific biomarkers, respectively. When applying the WoE approach, the cell-specific biomarkers in hepatocyte and cardiomyocyte differentiation are given higher weight compared to the germ-layer specific biomarkers and morphology/functionality. For the neural differentiation, both *PAX6* and *NESTIN* are given equal weight as they are considered cell-specific markers. In this case, both biomarkers are given higher weight than the morphological read-out.

Every measurement/observation is assigned a score (positive, negative, or equivocal). Obtained results from each individual read-out are weighed to provide an overall lineage call (Supplementary Table S3). Subsequently, all lineage calls are combined into an overall teratogenicity prediction (positive, negative, or equivocal) (Supplementary Table S4). In the ReproTracker assay, a compound is identified as a teratogen if it receives a positive score in at least one of the trilineage differentiation assays (cardiomyocyte, hepatocyte, or neural differentiation). If a compound induces an equivocal result—meaning the response is not conclusive enough to be classified as either positive or negative—in one or more lineages without affecting the other lineages, the overall response is considered equivocal. If neither biomarker expression levels nor morphology/functionality are affected in any of the differentiation assays, the response is considered negative.

The Lowest Observed Adverse Effect Level (LOAEL) defines the lowest tested concentration where, following teratogenicity classification, at least one of the biomarkers showed a significant decrease in expression levels. The No Observed Adverse Effect Level (NOAEL) indicates the highest tested concentration where none of the biomarkers demonstrated a significant reduction in expression.

### 2.9 Data acceptance and statistical analyses

Data acceptance for dose-range finding experiments was based on the response to positive and negative assay controls, and compound solubility and cytotoxicity. Exposure to the positive control (5-fluorouracil) should induce a cytotoxic response (reducing cell survival below 80%) at concentrations starting from 1.25 to 2.5 μM, while exposure to the negative control (saccharin) exposure should not induce any cytotoxicity in hiPSCs. Moreover, compound concentrations demonstrating precipitation in vehicle or in cell culture medium were excluded from the data analysis. Concentrations of compound(s) inducing cytotoxicity and therefore reducing cell survival below 80% were excluded from further testing in differentiation assays.

Data acceptance for every differentiation experiment was determined based on expected cell morphology/functionality of differentiating cells in solvent-exposed cultures, biomarker expression levels in solvent-exposed cultures in line with historical control databases, expected responses of differentiating cultures to the positive and negative assay controls, and compound cytotoxicity in each differentiation assay.

Morphologically, cardiomyocytes are expected to group in fiber-like 3D structures that distribute throughout the entire plate. Aside from morphology, an additional criterion for cardiomyocyte differentiation in each experimental setup is for the contracting mature cardiomyocyte cultures exposed to solvent controls, to reach 75%. Hepatocytes are expected to exhibit a cobblestone-like morphology, characterized by a distinct polygonal cell shape and well-defined cell boundaries. Neural rosettes are recognized by well-defined cell boundaries and multi-layered cultures. Functional/morphological assessment was completed independently from biomarker data analyses. Morphology scores were confirmed by a second person. Failure to observe the anticipated cell morphology/functionality throughout differentiation would result in invalidation of the experiment. Disruption in the morphology of cardiomyocytes is described as a lack of 3D-fibre-like structures in the cultures as well as a clear lack of contractions. Morphologically aberrant hepatocytes are characterized by a lack of polygonal structures with smaller cytoplasm that in the control cultures and the formation of dark cell-dense structures, commonly indicative of clumps of dead cells with big empty spaces between cell colonies. Finally, affected neural cultures are characterized by the presence of elongated or enlarged cell structures forming “star-like” shapes or cells with big extracellular spaces and lack of a clear multilayered organization. For data acceptance, functional/morphological changes of the cells in the presence of positive and negative assay controls (thalidomide and saccharin in cardiomyocyte and hepatocyte differentiation, and retinoic acid and saccharin in neural differentiation, respectively), included in each experiment, should be identified to ensure differentiations are successful.

Results are expressed as ± standard deviation (SD). Statistical analyses were performed using GraphPad Prism Software Version 10 (California, United States).

## 3 Results

### 3.1 Directed differentiation of hiPSCs into neural rosette-like cells

**FIGURE 1:**
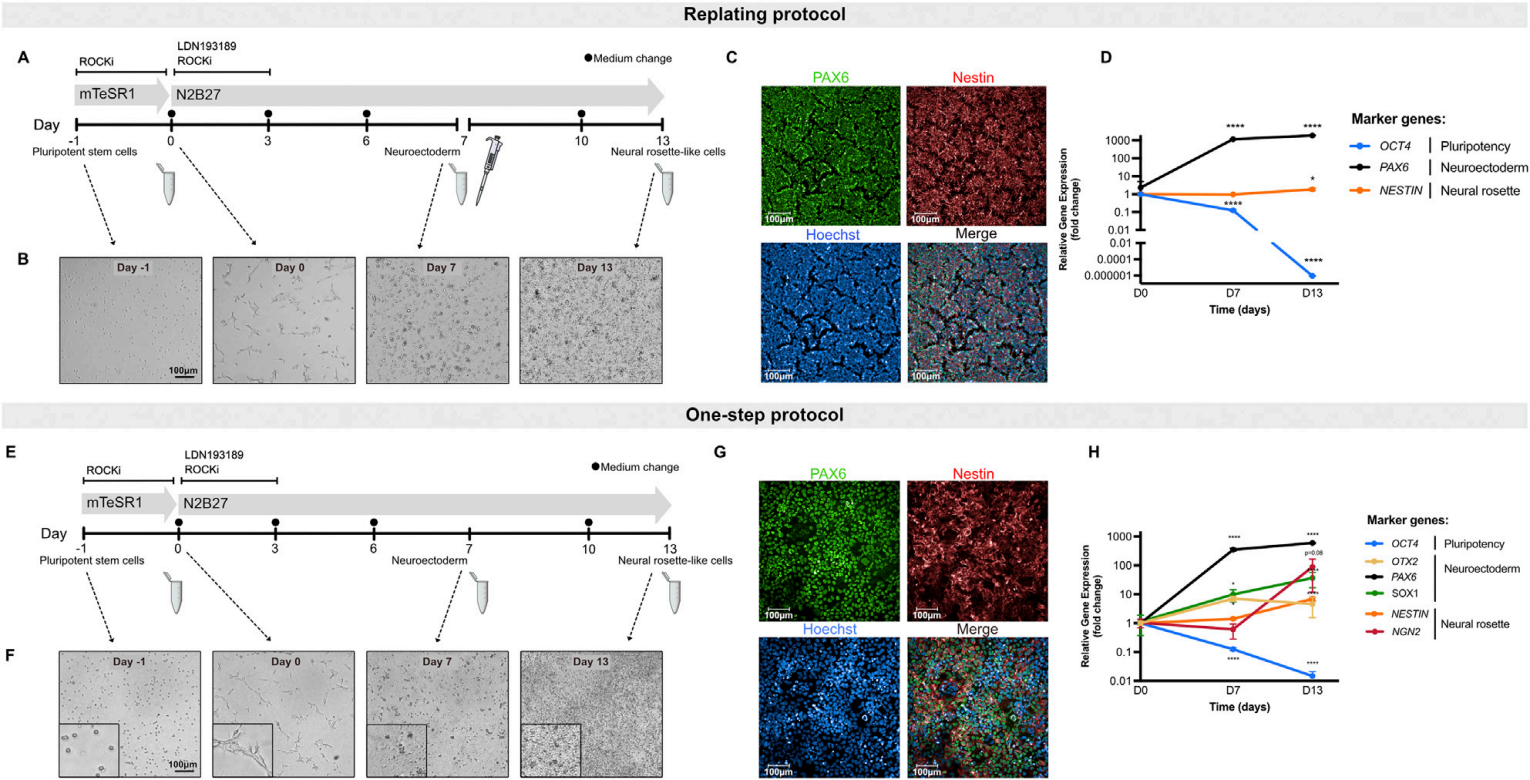
Differentiation of hiPSCs toward neural rosette-like cells. **(A)** Schematic representation of the differentiation of hiPSCs into neural rosette-like cells following replating protocol. Cells were replated on D7 of differentiation (pipette symbol). Samples were collected at D0, D7 and D13 (Eppendorf tube) for downstream processing. **(B)** Representative brightfield images of cells throughout differentiation following the replating protocol. Scale bar = 100 µm. **(C)** Representative immunofluorescence images of neural rosette-like cells at D13 following the replating protocol. Cells were stained for markers PAX6 (green) and NESTIN (red) with Hoechst counterstain for nuclei (blue). Scale bar = 100 µm. **(D)** Relative gene expression data of hiPSCs during neural rosette differentiation following replating protocol. Shown are the pluripotency marker *OCT4*, neuroectodermal markers *PAX6* and the neural rosette biomarker *NESTIN*. **(E)** Schematic representation of the differentiation of hiPSCs into neural rosette-like cells following one-step protocol. Samples were collected at D0, D7 and D13 (Eppendorf tube) for downstream processing. **(F)** Representative brightfield images of cells throughout differentiation following the one-step protocol. Scale bar = 100 µm. **(G)** Representative immunofluorescence images of neural rosette-like cells at D13 following the one-step protocol. Cells were stained for markers PAX6 (green) and NESTIN (red) with Hoechst counterstain for nuclei (blue). Scale bar = 100 µm. **(H)** Relative gene expression data of hiPSCs during neural rosette differentiation following one-step protocol. Shown are the pluripotency marker *OCT4*, neuroectodermal markers *OTX2*, *PAX6* and *SOX1* and the neural rosette biomarkers *NESTIN* and *NGN2*. Data represent mean ± SD from 3 biological replicates (*P < 0.05, **P < 0.01, ***P < 0.001, ****P < 0.0001, one-way ANOVA).

To develop an hiPSC-based *in vitro* assay for screening neurodevelopmental toxicants, two methods were implemented and optimized: a previously described approach, hereafter referred to as the replating protocol (Fedorova, 2019) (Figure 1A), and an in-house adapted method, termed the one-step protocol (Figure 1E). The latter eliminates the need for cell passaging at D7, allowing for the direct differentiation of hiPSCs into neural rosette-like cells. Differentiation of hiPSCs into neural rosette-like cells was assessed by determination of PAX6-and NESTIN-positive cells (protein levels) at D13 (Figures 1C,G). Moreover, evaluation of differentiation efficiency was based on qualitative morphological analysis at D13 (Figures 1B,F) and biomarker gene expression assessment at D7 and D13 (Figures 1D,H).

Both protocols effectively induced neural rosette-like cells, as confirmed by immunofluorescent visualization of the neuroectodermal marker PAX6 (localized in the nucleus) and the general neural stem cell marker NESTIN (a cytoskeletal protein) by day 13 (Figures 1C,G). In each experimental setup, at least 70% of the differentiated control cultures were positive for PAX6, demonstrating the high efficiency of both differentiation protocols in generating neural rosette-like cells (Supplementary Figure S1).

To quantitatively assess the efficiency of stem cell differentiation into neural rosette-like cells, a set of biomarkers, indicative of different stages of neural development, were selected (Figures 1D,H). Gene expression of *PAX6* was induced by D7 and its expression increased throughout the differentiation period. Moreover, *OTX2*, one of the early homeobox genes expressed in neuroectoderm, was also upregulated at D7, confirming the commitment of hiPSCs towards neuroepithelial progenitors in culture at D7 and the continuation towards neural rosette-like cells (Figure 1H). *SOX1*, another neuroectodermal marker, was also upregulated both at D7 and D13 (Figure 1H). The neural rosette markers *NESTIN* and *NGN2* were markedly expressed at D13, indicating proper differentiation into neural rosette-like cells (Figures 1D,H). Of note, *NESTIN* was already expressed in undifferentiated hiPSCs and its upregulation at D13 was less pronounced compared to *NGN2* (Figures 1D,H). Additionally, the expression of the pluripotency gene *POU5F1* (*OCT4*) was significantly downregulated during differentiation (Figures 1D,H). Altogether, these findings demonstrate that this system enables robust and reproducible differentiation into neural rosette-like cells, with both protocols exhibiting comparable regulation of gene expression and protein levels.

### 3.2 Assessment of neurodevelopmental toxicity at transcript and protein level

Next, the efficiency of both protocols in detecting neurodevelopmental toxicity potential of different compounds was assessed. For this purpose, two neurodevelopmental toxicants (retinoic acid and tacrolimus) as well as a negative control (saccharin) were selected (Aschner et al., 2017), and their impact on hiPSCs differentiation into early ectoderm and neural-like cells was studied. Assessment of the compounds’ impact on stem cell differentiation was performed at non-cytotoxic concentrations, as highly cytotoxic concentrations of compounds would disturb proper stem cell differentiation while not providing relevant information about their potential teratogenic properties (Lauschke et al., 2020; Kameoka et al., 2014; Waldmann et al., 2014). Thus, to determine cytotoxicity of the test compounds, undifferentiated hiPSCs were exposed to 20 consecutive concentrations at 2-fold dilutions for 7 days. The lowest concentration that induced a maximum of 20% cytotoxicity in the concentration range finding experiment was applied as top concentration for the differentiation experiments. Based on toxicity profile, the top concentrations for retinoic acid, tacrolimus, and saccharin were 5 µM (Supplementary Figure S2), 7.81 µM, and 250 µM (as described in our previous study) (Jamalpoor et al., 2022), respectively.

The compounds were tested in both the replating and the one-step protocols. Due to the nature of the protocols, teratogenicity prediction of the compounds was performed based on quantification of PAX6 and NESTIN-positive cells at D13 in the replating protocol or on a significant reduction in the biomarker gene expression at D7 and D13, as well as qualitative morphological analysis at D13 in the one-step protocol.

**FIGURE 2:**
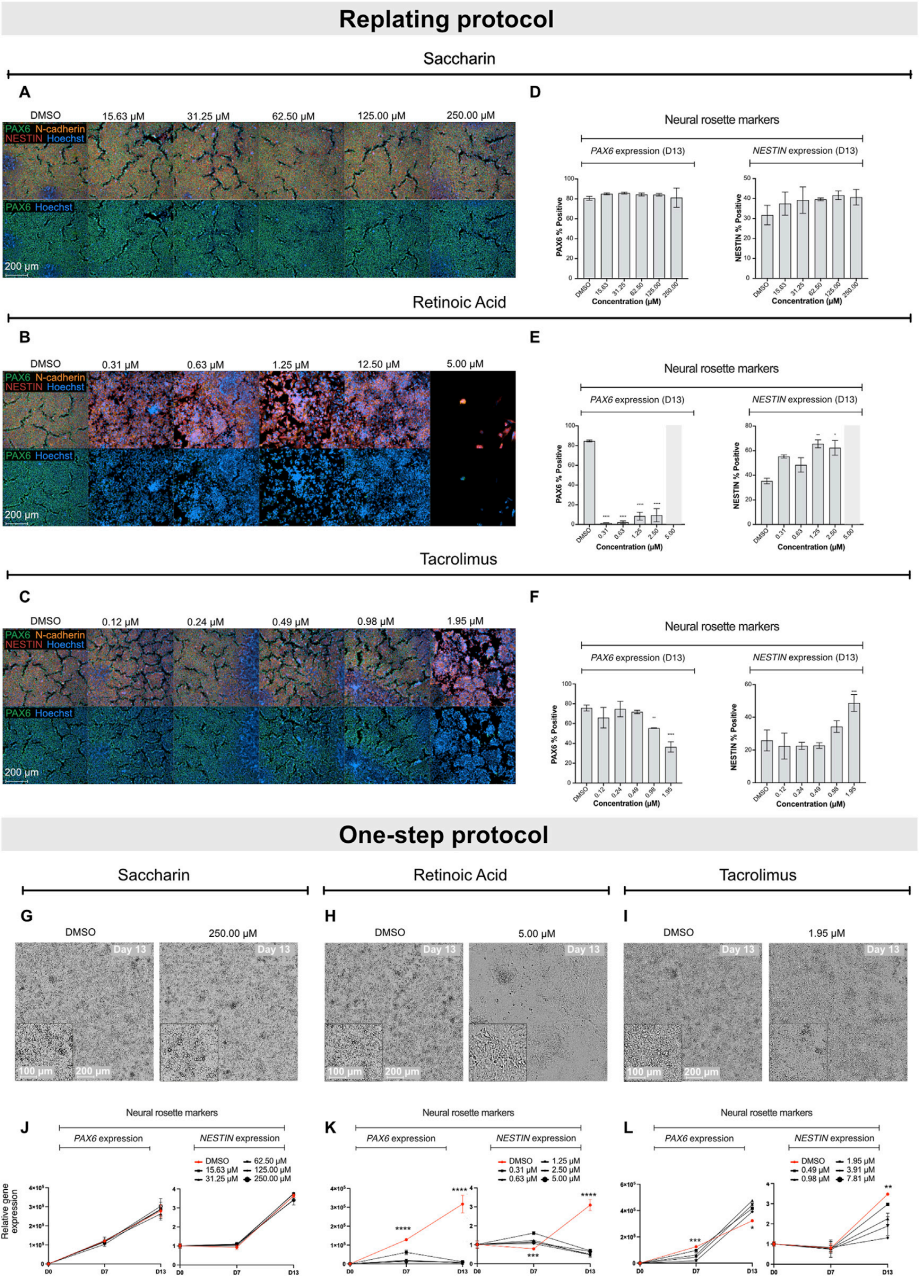
Effect of saccharin, retinoic acid and tacrolimus on the differentiation of hiPSCs into neural rosette-like cells. Representative immunofluorescence images of neural rosette markers PAX6 (green), NESTIN (red) and N-cadherin (orange) with Hoechst counterstain for nuclei at D13 after exposure to increasing concentrations of saccharin **(A)**, retinoic acid **(B)** and tacrolimus **(C)**. Scale bar = 200 µm. **(D–F)** Immunofluorescence quantification of biomarkers PAX6 and NESTIN positive cells from data shown in A, B and C, respectively. Data represent mean ± SD from 3 biological replicates (*P < 0.05, **P < 0.01, one-way ANOVA). **(G–I)** Representative brightfield images of neural rosette-like cells at D13 exposed to DMSO and the highest tested non-cytotoxic concentrations of saccharin, retinoic acid and tacrolimus, respectively. Scale bar = 100–200 µm. Relative gene expression of *PAX6* and *NESTIN* in cells exposed to increasing concentrations of saccharin **(J)**, retinoic acid **(K)**, and tacrolimus **(L)**. Data represent mean ± SD from 3 biological replicates. Statistical comparison depicted between vehicle control and top tested concentration (*P < 0.05, **P < 0.01, ***P < 0.001, ****P < 0.0001, one-way ANOVA).

Exposure of differentiating hiPSCs to saccharin had no effect on the protein levels or expression of the selected biomarker genes, nor on the morphology in both protocols (Figures 2A,D,G,J; Supplementary Figure S3A). Retinoic acid, in the replating protocol, significantly decreased PAX6 protein levels at all non-cytotoxic concentrations and disrupted NESTIN localization with increasing NESTIN protein levels (Figures 2B,E). Concordantly, in the one-step protocol, increasing concentrations of retinoic acid led to a downregulation of *PAX6* and *OTX2* genes expression at both D7 and D13 compared to vehicle controls (Figure 2K; Supplementary Figure S3B). Additionally, retinoic acid downregulated *NESTIN* expression only at D13, while it increased *NESTIN* gene expression at D7 compared to vehicle controls. Retinoic acid also disrupted the morphology of neural rosette-like cells (Figure 2H). Notably, in the replating protocol, the effect of retinoic acid was more pronounced; at the highest tested concentration (5 µM), it induced complete cell death opposed to the effects seen in the one-step protocol. Cell replating has been suggested to increased cellular stress and a transient increase in cytotoxicity responses (Reiners et al., 2000), which suggests that the replating protocol might make cultures more sensitive to chemical exposure. Tacrolimus demonstrated a similar effect to that of retinoic acid, leading to a concentration-dependent decrease in PAX6 protein levels, and affecting NESTIN localization. Moreover, tacrolimus, at the highest tested concentration, also increased NESTIN protein levels (Figures 2C,F). In the one-step protocol, increasing concentrations of tacrolimus, unlike retinoic acid, led to a downregulation of *PAX6* only at D7, while increasing *PAX6* gene expression at D13 (Figure 2L). Like retinoic acid, tacrolimus downregulated *NESTIN* expression only at D13, though the effect was less pronounced. Addition of tacrolimus also disrupted the morphology of neural rosette-like cells at D13 (Figure 2I). Of note, tacrolimus had no significant effect on the expression of *NGN2*, *OTX2* and *SOX1* genes (Supplementary Figure S3C **)**. These results highlight the dynamic regulation of differentiation markers and how these can be differentially affected by chemical treatment, reinforcing the importance of the two biomarkers (*PAX6* at D7 and *NESTIN* and D13) as key indicators of neurodevelopmental toxicity in this assay.

Altogether, the data indicate that both protocols are effective for teratogenicity prediction *in vitro*. The regulation of PAX6 at D7 showed a clear correlation between gene expression and protein levels. However, the regulation of NESTIN exhibited more complex dynamics. Teratogens such as retinoic acid and tacrolimus both caused a pronounced downregulation of *NESTIN* gene expression at D13, while absolute protein levels remained unchanged (or increased). In contrast, protein localization within the neural rosette structures was significantly altered. Since disruption of protein localization is difficult to quantify, assessing gene regulation provides a faster and more quantitative approach to evaluate compounds that affect neural cell development. Moreover, the replating protocol is more labor-intensive and less suitable for early-stage compound screening. As a result, the one-step protocol was incorporated into the ReproTracker assay for teratogenicity screening and was used as the standard protocol throughout the remainder of the study.

### 3.3 Miniaturization of the ReproTracker assay for higher throughput screening

To better position the assay for drug screening, ReproTracker was downscaled from a 24-well to a 96-well plate format. The 24- to 96-well plate optimization was divided into multiple steps, including: cell density optimization, morphology evaluation, biomarker kinetic assessment and validation.

Multiple densities were tested for the seeding of the experiments to maintain the same cell/cm^3^ as in the 24-well plate format and ensure optimal expression of the key biomarkers. The optimization led to a final seeding density of 5K cells per well for the neural differentiation, 14K cells per well for the cardiomyocyte differentiation, and 10K cells per well for the hepatocyte differentiation. During the optimization in 96-well plates, it was observed how the hepatocyte differentiation efficiency at D21 was reduced when compared to 24-well plates (Supplementary Figure S4A). Therefore, medium composition was re-assessed. For this purpose, two new conditions were compared to the standard medium in the 96-well plate differentiation, including addition of HepatoZYME medium (ThermoFisher Scientific) or hydrocortisone (Sigma-Aldrich), at D10 (Supplementary Figure S4B). Based on this optimization, the standard medium supplemented with hydrocortisone led to higher levels of *AFP* expression at the end of the differentiation and reduced the variation between independent samples when compared to the standard medium. Interestingly, using this new medium formulation hepatocyte-like cells showed an expected induction of *AFP* expression already at D14, allowing for the differentiation to be shortened from 21 to 14 days (Supplementary Figure S4B,E). Secondly, morphological and immunostaining evaluation of the cultures in the downscaled format did not suggest any morphological alterations in the 96-well plate compared to the 24-well plate (Supplementary Figures S4C and S4D). Moreover, kinetic regulation of the key biomarkers, assessed at the different stages of the differentiations were consistent compared to the 24-well plate system, as previously reported (Supplementary Figure S4E).

Finally, to ensure robustness of the new optimized method, a subset of previously tested compounds was evaluated in both formats (24- and 96- well plate) (Supplementary Table S5), and the results demonstrated that downscaling did not affect predictive capacity of the assay. Moreover, this miniaturization of the ReproTracker assay also enabled the use of robotic platforms–reducing compound volume requirements–and accelerated turnaround times, making the assay more practical for early-stage screening of developmental toxicity.

### 3.4 Applicability of the extended ReproTracker assay for *in vitro* detection of teratogenicity

To demonstrate the applicability of the extended ReproTracker assay following inclusion of the ectoderm lineage, we tested 19 different pharmaceuticals and chemicals based on an internal selection of well-known teratogens and non-teratogens, which had previously been assessed in the ReproTracker system, as well as 32 reference compounds listed in the ICH S5 (R3) guidelines (Table 1). After obtaining cytotoxicity profiles for each test compound (Reiners et al., 2000) (Supplementary Table S6), a total of 5 consecutive concentrations were selected to continue the trilineage differentiation assays.

**FIGURE 3:**
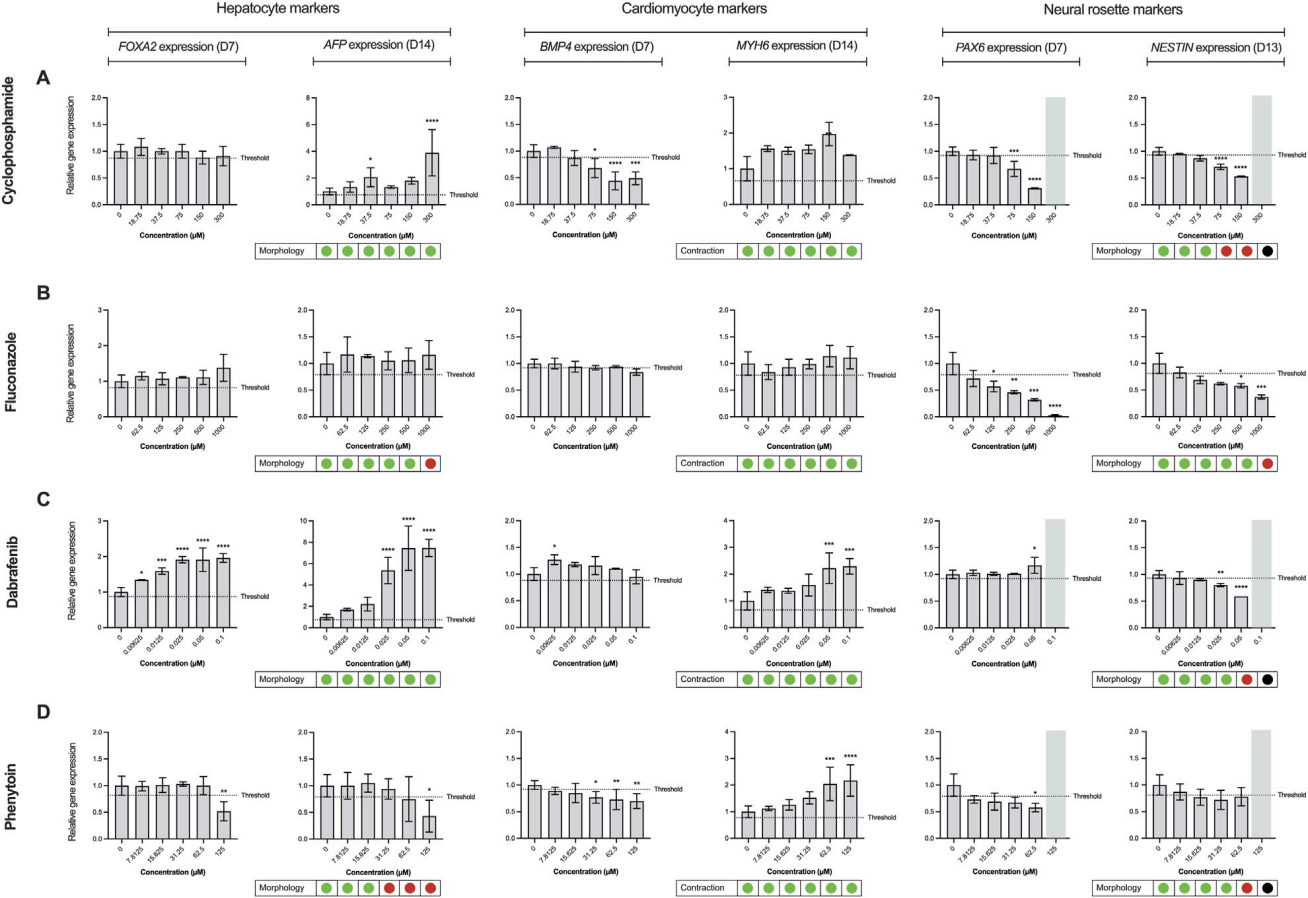
Effect of reference compounds in trilineage differentiation of the ReproTracker assay. Alterations in morphology, cardiac contraction ability, and gene expression patterns of *FOXA2* (endodermal marker), *AFP* (hepatocyte-specific marker), *BMP4* (mesodermal marker), *MYH6* (cardiomyocyte-specific marker), *PAX6* (neuroectodermal marker) and *NESTIN* (neural rosette biomarker) upon exposure to several upon exposure to in vivo teratogens cyclophosphamide **(A)**, fluconazole **(B)**, dabrafenib **(C)** and phenytoin **(D)**. Red dots indicate the test compound stopped cardiac contractability of cardiomyocytes or disrupted hepatocyte/neural rosette morphology at the end of differentiation, compared to vehicle controls. Green dots indicate the test compound had no effect on the cardiomyocyte beating or morphology of hepatocytes/neural rosette-like cells at the end of differentiation, compared to vehicle controls. Black dots, as well as the marked grey area, represent data that were excluded from the analysis due to observed cytotoxicity during the differentiation process upon exposure to the test compound. Expression of the biomarkers were measured using qRT-PCR, normalized to control (vehicle-treated) cells at day 0. Data are expressed as the mean ± SD. Statistically significant compared to control condition (*p < 0.05, **p < 0.01, ***p < 0.001, ****p < 0.0001 (one-way ANOVA followed by Dunnett’s multiple comparisons test).

Here, it was first evaluated whether inclusion of the ectoderm lineage would enhance the predictive capability of the ReproTracker assay in identifying human teratogens. The correct teratogenicity prediction of 9 out of 51 chemicals (busulfan, clarithromycin, cyclophosphamide, dabrafenib, dexamethasone, fluconazole, mirex, ribavirin, and topiramate) was solely achieved through the incorporation of neural differentiation into the initial ReproTracker assay (Table 1; Figure 3). Most of these compounds are known to induce (neuro) developmental toxicity *in vivo* (Braillon, 2023; Ferm et al., 1978; Adams et al., 1990; Al-Musawi and Jaber, 2022; Chaube et al., 1968; Bandettini Di Poggio et al., 2011; Ohira et al., 2013; Challine et al., 1990; Xiao, 2007). Cyclophosphamide, for instance, is a chemotherapy drug known to be teratogenic in humans (Mirkes, 1985; Leyder et al., 2011), and has been associated with neurodevelopmental toxicity (Xiao et al., 2007; Ibrahim et al., 2024). In ReproTracker, cyclophosphamide had no significant effect on the expression patterns of biomarker genes specific for hepatocytes and cardiomyocytes, nor on their morphology/functionality. It only downregulated the expression of the mesodermal marker *BMP4*. However, in the neural differentiation assay, cyclophosphamide exposure led to a concentration-dependent downregulation of *PAX6* and *NESTIN* expression at D7, and D13, respectively. Exposure to cyclophosphamide also disrupted the morphology of neural rosette-like cells (Figure 3A). Like cyclophosphamide, fluconazole, a broad-spectrum antifungal drug linked to neurodevelopmental defects (Budani et al., 2021), only affected neural differentiation in the ReproTracker assay. Exposure of differentiating cells to fluconazole, decreased expression of both *PAX6* and *NESTIN* in a dose-dependent manner at D7, and D13, respectively (Figure 3B). *In vivo* teratogens dabrafenib and phenytoin are yet other examples where the addition of the neural lineage allowed for correct teratogenicity prediction. Dabrafenib downregulated *NESTIN* expression at D13, without affecting *PAX6* expression at D7 (Figure 3C), while phenytoin downregulated the expression of *PAX6* at D7 without affecting *NESTIN* expression at D13 (Figure 3D), emphasizing the importance of both biomarkers for teratogenicity prediction in the ReproTracker system. Both chemicals, at the highest tested non-cytotoxic concentration, also disrupted neural rosette morphology.

Moreover, the addition of the neural differentiation to the ReproTracker portfolio enhanced assay sensitivity in predicting compound teratogenicity. For example, imatinib–previously identified as a developmental toxicant based on its effects on hepatocytes (Jamalpoor et al., 2022) – impaired neural differentiation at a much lower concentration (Table 1). Inclusion of the neural differentiation readout reduced the LOAEL for imatinib from 31.3 µM to 0.1 µM, aligning with its clinically relevant human Cmax (1.5 µM) (R3 I, 2021; Schulz et al., 2020; Hensley and Ford, 2003). Similarly, diltiazem, initially assigned a LOAEL of 31 µM based on combined effects on cardiomyocytes and hepatocytes (Jamalpoor et al., 2022), showed a reduced LOAEL of ≤7.8 µM with the inclusion of neural differentiation assay (Table 1), enabling detection of its teratogenicity at concentrations relevant to its human Cmax (0.06–0.3 µM) (R3 I, 2021; Schulz et al., 2020; Ariyuki, 1975).

Overall, the ReproTracker assay correctly predicted the teratogenic potential of 44 out of the 51 compounds tested in this study. Three out of five misclassified teratogens were identified as DNA modifying agents, namely, 5-fluorouracil, methotrexate and methylmercury. All three compounds exhibited a highly cytotoxic profile which led to very low concentrations being tested in the assay (1, 0.1, and 0.3 μM, respectively) (Table 1). Vildagliptin and hydrochlorothiazide, two *in vivo* non-teratogens, were falsely labeled as developmental toxicants in ReproTracker. Of note, vildagliptin impaired neural differentiation at concentrations much higher (200-fold) than its human Cmax (0.6–1 μM) (R3 I, 2021; Schulz et al., 2020). Altogether, based on the current dataset of 51 compounds, the inclusion of neural rosette differentiation improved the ReproTracker assay accuracy (from 72.55% to 86.27%) and sensitivity (from 67.50% to 87.50%). These findings highlight how multi-lineage differentiation in ReproTracker expands biological coverage and enhances teratogenicity prediction. The strong overlap between ReproTracker results and *in vivo* predictions underscores the assay’s consistently high performance compared to its original version (Jamalpoor et al., 2022).

## 4 Discussion

### 4.1 Establishing new NAMs for a wider biological coverage *in vitro*

Developing advanced human-based *in vitro* models capable of recapitulating key biological processes during sensitive embryo developmental stages is imperative for accurately predicting the developmental toxicity of new drugs and chemicals. We developed ReproTracker, a trilineage differentiation assay that follows the differentiation of hiPSCs into hepatocyte-, cardiomyocyte-, and now neural-like cells. This expansion broadened the spectrum of teratogenic agents detectable by ReproTracker–including those affecting neurodevelopment–while maintaining high sensitivity and specificity.

Neurodevelopmental toxicants can disrupt the function of the central and/or peripheral nervous system, potentially leading to various pathologies or even death (Rock and Patisaul, 2018; Tamm and Ceccatelli, 2017). These toxicants include pharmaceuticals, cosmetics, and environmental pollutants such as pesticides (Rock and Patisaul, 2018). In this study, the incorporation of hiPSCs differentiation towards the neuroectoderm lineage significantly enhanced the biological relevance of ReproTracker in detecting potential neurodevelopmental toxicants.

### 4.2 The role of PAX6 and NESTIN regulation in assessing neurodevelopmental toxicity

The paired type homeobox 6 (*PAX6*) is a transcription factor that plays a key role in the embryonic central nervous system development (Curto et al., 2014). *PAX6*, commonly expressed in neuroepithelial progenitors, is heavily involved in neuronal commitment (Curto et al., 2014; Bel-Vialar et al., 2007) and it has been suggested to be the earliest determinant for neuroectoderm in human development (Li et al., 2005; Zhang, 2010). In ReproTracker, upregulation of *PAX6* expression at D7 indicates the commitment of hiPSCs towards neuroepithelial progenitors in culture (Fedorova et al., 2019). *OTX2*, another neuroectodermal biomarker essential for the induction of fore-brain differentiation (Hoch et al., 2015), was also upregulated in the neural rosette-like cells. Moreover, regulation of *PAX6* is key for the upregulation of the expression of nuclear factor *NGN2* in neural progenitors (Bel-Vialar et al., 2007). Mutual regulation of *PAX6* and *NGN2* is crucial for the cells to exit their intermediate state and exit the cell cycle and continue towards neural differentiation (Bel-Vialar et al., 2007). Another crucial biomarker for neuroectoderm commitment is *SOX1*, which has also been described to favor neuroectodermal commitment and interacts with *PAX6* to regulate this state and lead to further differentiation (Suter et al., 2009). Another key biomarker in neural development is the intermediate filament protein *NESTIN* (Suzuki et al., 2010). This neural stem/progenitor cell marker is highly expressed in neuroepithelial progenitors and quickly downregulated in mature neurons and glial cells (Suzuki et al., 2010; Dahlstrand et al., 1995). All the above biomarkers are crucial for correct *in vitro* differentiations of hiPSCs towards structures known as neural rosettes. These structures are formed by radial groups of cells with an empty lumen and are thought to mimic the early development of the neural tube (Miotto et al., 2023; Wilson and Stice, 2006).

One key event observed following treatment with several neurodevelopmental toxicants is the disruption of neural rosette formation. This event includes reduction in the number and/or alteration in the structure of cells differentiating towards other neural cell types (Dreser et al., 2020). *PAX6* has been described as one of the most vulnerable genes in response to fetal alcohol exposure in *Xenopus* embryos (Peng et al., 2004). Moreover, it has been found to be a key regulator in human eye development, with nonsense and missense mutations leading to loss-of-function and congenital eye malformations (Hanson, 2003). Additionally, loss of *PAX6* expression in different animals is known to induce eye malformations, highlighting the importance of *PAX6* regulation in key developmental processes (Callaerts et al., 2001; Hill et al., 1991). On the other hand, *NESTIN* expression has been linked with cortical cells proliferation, which highlights its importance for cell differentiation (Bernal and Arranz, 2018). Downregulation of *NESTIN* has been correlated to impairments in cell proliferation (Xue and Yuan, 2010). Therefore, we proposed both genes as key regulators and biomarkers to identify developmental toxicity in the ReproTracker assay.

### 4.3 Assessment of neural biomarkers at different developmental stages

The selection of *PAX6* as a key biomarker at D7 rather than D13 for teratogenicity prediction in ReproTracker was based on different biological and technical aspects. First, *PAX6* has been previously hypothesized to be the earliest biomarker determining neuroectodermal fate *in vitro* (Zhang et al., 2010). Moreover, *PAX6* expression is significantly upregulated between D0 and D7 of differentiation, indicating the changes in cell fate from pluripotency towards neuroectoderm. Using ReproTracker, we validated the correlation between *PAX6* and *NESTIN* alterations *in vitro,* as a response to teratogenic compounds, and the corresponding *in vivo* teratogenicity of the compound. To this end, we were able to correlate a downregulation of *PAX6* and/or *NESTIN* expression at D7 and D13, respectively, within the neural differentiation assay, to the teratogenic potential of the compounds. Using this data set, we observed that, out of the 16 compounds affecting the neural differentiation in ReproTracker, 10 led to a significant downregulation of both *PAX6* and *NESTIN* expression at D7 and D13, respectively. Four compounds showed a clear effect on the expression of *PAX6* at D7 but not of *NESTIN* at D13, while exposure to two of them led to only a downregulation of *NESTIN* at D13 with no clear effect on *PAX6*. Moreover, some compounds led to a decrease of *PAX6* expression at D7, but an increase at D13. This initial downregulation of *PAX6* at D7 upon chemical exposure suggests a delay in neuroectoderm induction (Zhang et al., 2010). However, by D13 the induction of *PAX6*, might suggest a non-canonical compensatory mechanism to induce neurogenesis (Pinson et al., 2006). These results highlight the importance of *PAX6* and *NESTIN* expression in the different stages of the differentiation and potentially explain the adverse effects that might stem from inhibiting the processes that regulate both biomarkers *in vitro*.

### 4.4 ReproTracker as a tool for developmental toxicity predictions

In this study, ReproTracker was validated against a large set of compounds, including those from the ICH S5 (3R) reference list and several other teratogens and non-teratogens. Overall, based on the current dataset of 51 compounds, the ReproTracker assay correctly predicted the teratogenic potential of chemicals with high accuracy (86.27%) and sensitivity (87.50%). A subset of DNA-modifying agents tested– 5-fluorouracil, methotrexate, and methylmercury–exhibited highly cytotoxic profiles, limiting their highest testable concentration to 0.1–1 µM. Although these compounds are known *in vivo* teratogens, they could not be classified as positives in ReproTracker. High cytotoxicity has been suggested to mask potential teratogenic effects, complicating compound assessment (Xing et al., 2017). Consequently, these agents were classified as false negatives in the ReproTracker assay. Additionally, methanol and trimethadione were mislabeled as false negatives in the assay. Although teratogenic effects of methanol have been detected in rodent studies (Hansen et al., 2005), this compound is not considered to be a reproductive or developmental toxicant in humans due to insufficient human data (NTP-CERHR Expert Panel et al., 2004). Trimethadione, an anticonvulsant, has been shown to be highly teratogenic in mice (Brown et al., 1979) and is a suspected human teratogen (Feldman et al., 1977; Midha et al., 1979).

Regarding the identification of false positives in ReproTracker, the C_max_ of vildagliptin in humans has been reported to range between 0.6 and 1 µM (R3 I, 2021; Schulz et al., 2020), whereas the LOAEL in ReproTracker was approximately 200-fold higher (125 µM). Despite the absence of adequate human data on pregnancy in combination with the use of vildagliptin, this drug is not recommended to be taken during pregnancy unless benefits outweigh potential risks. The teratogenicity prediction of hydrochlorothiazide, which was previously detected as a positive in the cardiomyocyte and hepatocyte differentiation assays of ReproTracker (Jamalpoor et al., 2022), did not change by the addition of the neural differentiation assay. However, its LOAEL in ReproTracker (62.5 µM) was approximately 600-fold higher than the compound’s described C_max_ (0.1–0.2 µM). A potential explanation of false positives could be that compounds may activate stress pathways or signaling cascades that indirectly affect differentiation without being teratogenic.

**FIGURE 4:**
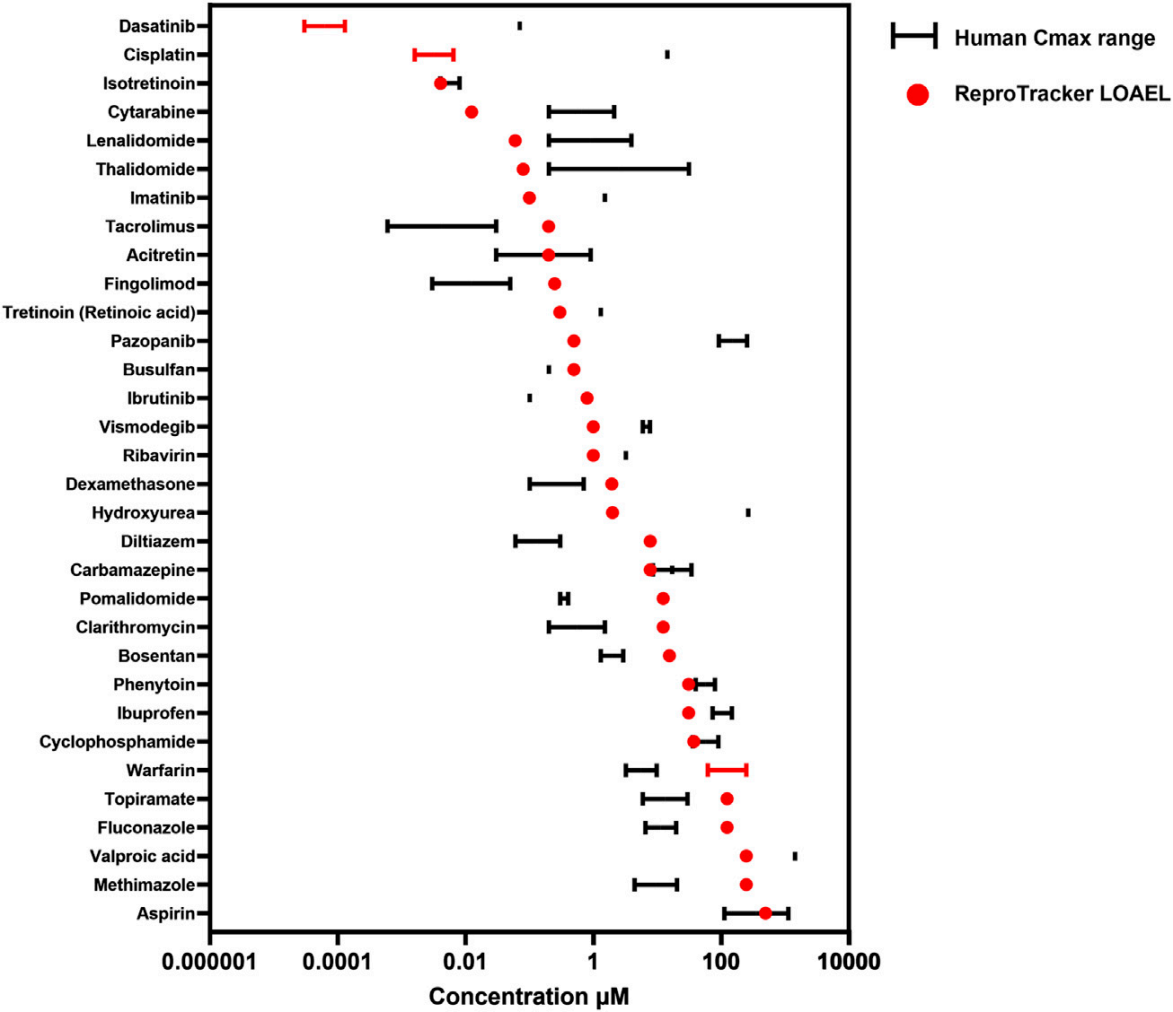
Correlation between the lowest observed adverse effect level (LOAEL) of teratogens in ReproTracker and their corresponding human therapeutic Cmax as obtained from the literature. Values are represented as a single point when only one value was available or as a range depending on data availability.

The ICH S5 (3R) guidelines aim to set the way for the acceptance and validation of alternative models in DART testing by optimizing the requirements and improving the regulatory confidence in NAMs. A key requirement for these NAMs is the ability to identify teratogenic compounds at clinically relevant concentrations, ideally aligning with *in vivo* exposures observed in humans (C_max_). Following the identification of human developmental toxicants using ReproTracker, we assessed the clinical relevance of the concentrations flagged as potentially teratogenic by examining their correlation with their corresponding human therapeutic C_max_ values. To this end, we compared the lowest observed adverse effect level (LOAEL) values obtained in ReproTracker–assuming complete cellular uptake of the administered concentration–with the therapeutic C_max_ of each positive compound (Figure 4).

In general, ReproTracker identified the teratogenic potential of 19 out of 35 known *in vivo* teratogens (dasatinib, cytarabine, lenalidomide, imatinib, retinoic acid, pazopanib, ribavirin, vismodegib, cisplatin, hydroxyurea, valproic acid, isotretinoin, methotrexate, acitretin, thalidomide, carbamazepine, phenytoin, cyclophosphamide, and aspirin) at concentrations equal to or below their respective human therapeutic C_max_ (Figure 4). In cases where the predicted LOAEL exceeded the C_max_, the values still remained within a 10- to 40-fold range, indicating reasonable proximity to clinically relevant exposure levels. While the LOAEL of diltiazem, fingolimod, pomalidomide and tacrolimus was higher than the respective C_max_, this outcome was primarily constrained by the lowest tested concentration in the assay. Testing lower concentrations could have yielded lower LOAEL values approaching their respective C_max_. Of note, *in vitro* to *in vivo* extrapolation (IVIVE) is warranted to better translate the *in vitro* bioactive concentrations to exposures that would be equal to or predicted to result in plasma concentrations. ReproTracker data has been used previously in combination with physiologically based pharmacokinetic (PBPK) models to derive human equivalent doses (HEDs) (Moreau et al., 2023). ReproTracker’s LOAELs for thalidomide and carbamazepine were used to calculate their corresponding HEDs (Moreau et al., 2023). In both cases, the calculated HEDs were identified to be equal or below their respective human teratogenic concentrations. These results illustrate the potential of the combined use of ReproTracker data and PBPK modeling to predict HEDs without the need for animal models.

## 5 Conclusion

The ReproTracker assay marks a major advancement toward animal-free testing approaches, offering promising potential to revolutionize teratogenicity assessments across the pharmaceutical, cosmetic, and chemical sectors. Our study demonstrates that incorporating multi-lineage differentiation broadens its biological scope and improves its accuracy of teratogenicity predictions, establishing ReproTracker as a leading NAM for developmental toxicity evaluation.

## Data Availability

The original contributions presented in the study are publicly available. This data can be found here: 10.5061/dryad.1c59zw48q.
