# Supplementary material for: Implementing a trilineage differentiation in the ReproTracker assay for improved teratogenicity assessment
Source: Front Toxicol. 2025 Sep 25;7:1645842. doi: 10.3389/ftox.2025.1645842 (PMC12508576; doi:10.3389/ftox.2025.1645842)
Supplement: Supplementary file 1 [file DataSheet1.pdf]

Supplementary material

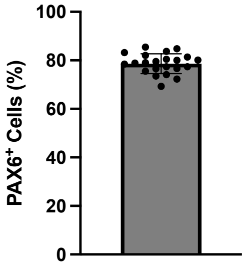

Supplementary Figure 1. Number of PAX6+ cells (in percentage) in the cultures following hiPSC differentiations. Data represent mean ± SD from 22 technical replicates across 2 biological replicates.

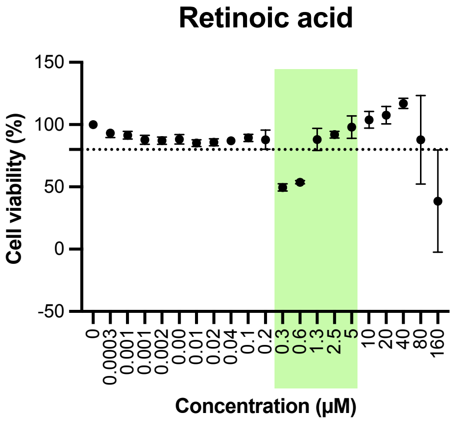

Supplementary Figure 2. Dose range finding experiments for retinoic acid. Data represent mean ± SD from 3 biological replicates.

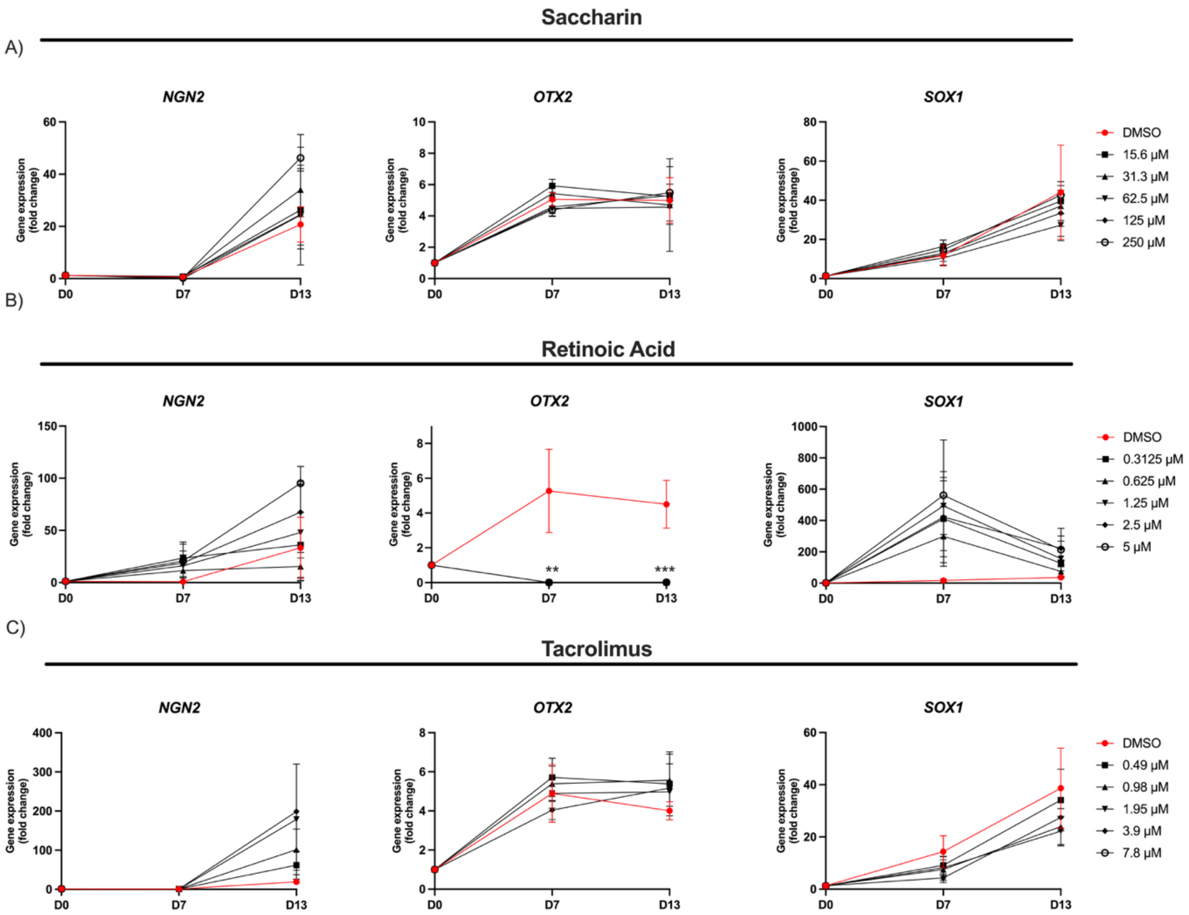

Supplementary Figure 3. Relative gene expression (fold change) of NGN2, OTX2 and SOX1 in cells exposed to increasing concentrations of saccharin (A), retinoic acid (B) and tacrolimus (C). Data represents mean ± SD from 2 biological replicates. Statistical comparison depicted between vehicle control and top tested concentration (*P < 0.05, **P < 0.01, ***P<0.001, one-way ANOVA).

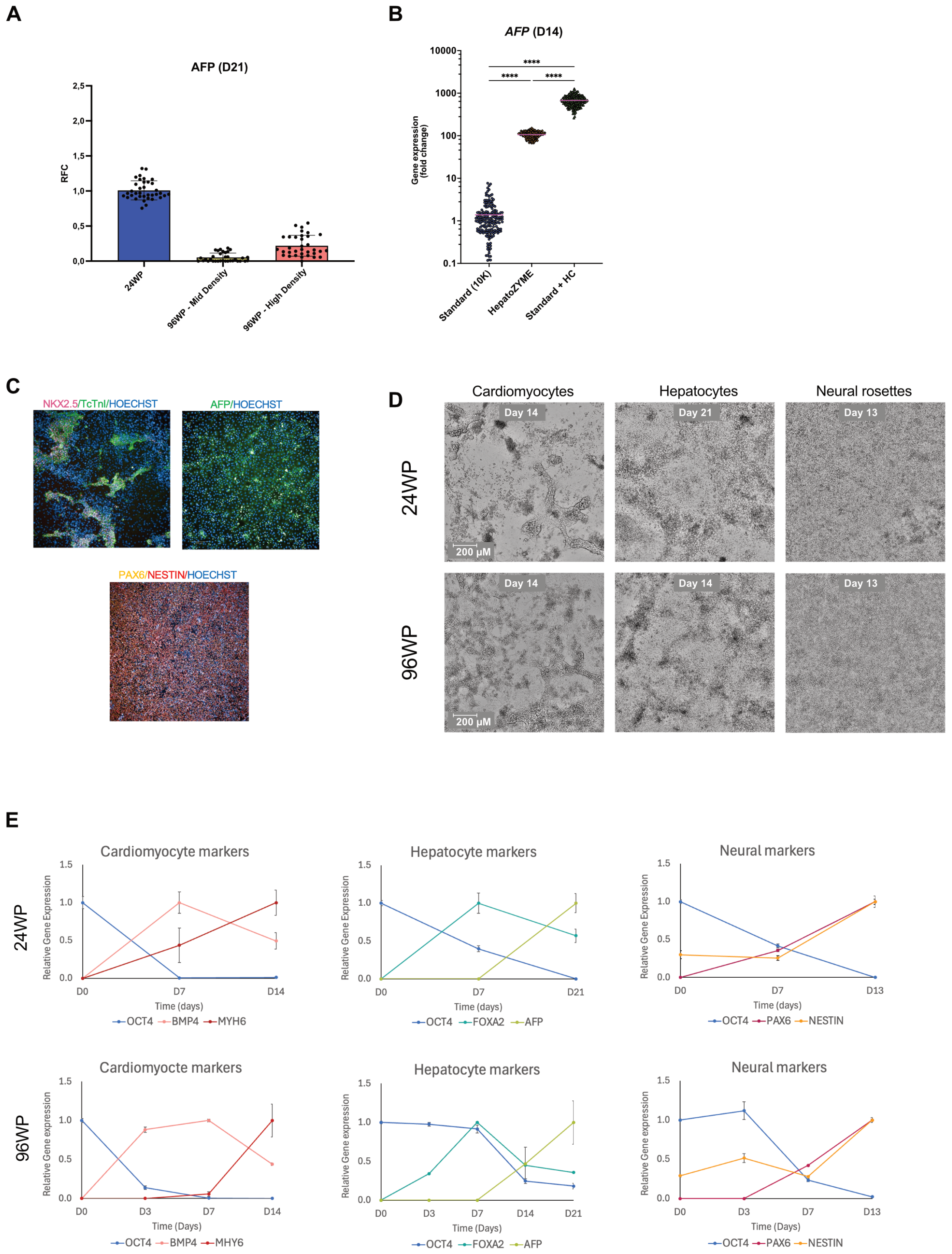

Supplementary Figure 4. Optimization of ReproTracker in 96 well plates. (A) Relative gene expression of *AFP* at D21 in hepatocytes differentiated in 24 well plates and 96 well plates at two different densities (8.5K and 10K). (B) Relative gene expression of *AFP* at day 14 in the 96-well plate system using using standard conditions, standard conditions using hepatoZYME medium from D10 to D14 and standard conditions supplemented with hydrocortisone (HC) from D10 to D14. Data represent mean ± SD from 183-189 technical replicates across 2 biological replicates (*P < 0.05, **P < 0.01, ***P<0.001, ****P<0.0001, one-way ANOVA). (C) Representative immunofluorescence images of cardiomyocytes, hepatocytes and neural rosette-like cells. Cells were stained for markers NKX2.5 (ref) and TcTN1 (green) with Hoechst counterstain for cardiomyocytes; AFP (green), for hepatocytes; and PAX6 (green) and NESTIN (red) with Hoechst

counterstain for nuclei (blue) for neural-rosettes.(D) Representative brightfield images of cardiomyocytes, hepatocytes and neural rosettes at the end of the differentiation in both 24- and 96-well plates. (E) Comparison between relative gene expression dynamics of OCT4, BMP4 and MYH6 in the cardiomyocyte differentiation; *OCT4*, *FOXA2* and *AFP*, in the liver differentiation; and *OCT4*, *PAX6* and *NESTIN* in the neural differentiation.

Supplementary Table 1: Primer and probes used for biomarker expression analysis.

Target gene	Assay ID	Reporter dye
GAPDH	Hs.PT.39a.22214836	Cy5
OCT4	Hs.PT.58.14494169.g	TAMRA
BMP4	Hs.PT.56a.2412580.g	FAM
MYH6	Hs.PT.58.40702029	SUN
FOXA2	Hs.PT.58.26032236	FAM
AFP	Hs.PT.56a.39466878	SUN
PAX6	Hs.PT.58.3002797	SUN
NESTIN	Custom assay	FAM
SybrGreen primers		
OTX2	Fw: GAG GTG GCA CTG AAA ATC AAC<br>Rv: GGC AGG TCT CAC TTT GTT TTG	
NGN2	Fw: CAGACATGGACTATTGGCAG<br>Rv: GGGACAGGAAAGGGAACC	
SOX1	Fw: TCC CCC GCG TGA ACT G<br>Rv: CAA GGC ATT TTG CGT TCA CA	
GAPDH	Fw: AAT CCC ATC ACC ATC TTC CA<br>Rv: TGG ACT CCA CGA CGT ACT CA	

Supplementary Table 2: Antibody overview.

Antibody	Species	Supplier	Cat. Number	Dilution (X)
Anti-PAX6	Mouse	Sigma (Atlas Antibodies)	AMAb91372	150
Anti-NESTIN	Rabbit	Sigma (Atlas Antibodies)	HPA006286	800
Anti-rN-Cadherin	Sheep	R&D Systems	AF6426	150
Anti-Mouse AlexaFluor 488	Donkey	Thermo Fisher Scientific	A21202	1000
Anti-Rabbit AlexaFluor 647	Donkey	Thermo Fisher Scientific	A32795	1000
Anti-Sheep AlexaFluor 555	Donkey	Thermo Fisher Scientific	A21436	1000

Supplementary Table 3. Lineage-specific weight of evidence approach. Possible scenarios of outcomes when combining the ReproTracker data (biomarker expression and morphology and/or functionality) for the cardiomyocyte and hepatocyte differentiations (A) and for the neural rosette-like cells (B). Results are assigned a score +, (+) or – in case of positive, equivocal or negative response.

(A)

**Cardiomyocyte / hepatocyte differentiation assay**			
Germ-layer specific marker	Cell-specific marker	Cell morphology/ functionality	Lineage call

(B)

**Neural differentiation assay**			
Germ-layer specific marker	Cell-specific marker	Cell morphology/ functionality	Lineage call

-	-	-	Negative
(+)	-	-	Negative
-	-	+	Negative
+	-	-	Negative
-	(+)	-	Equivocal
(+)	(+)	-	Equivocal
(+)	-	+	Equivocal
+	(+)	-	Equivocal
+	-	+	Equivocal
-	+	-	Positive
-	(+)	+	Positive
(+)	+	-	Positive
-	+	+	Positive
+	+	-	Positive
(+)	(+)	+	Positive
(+)	+	+	Positive
+	(+)	+	Positive
+	+	+	Positive

-	-	-	Negative
-	-	+	Negative
-	(+)	-	Equivocal
(+)	-	-	Equivocal
(+)	(+)	-	Equivocal
-	+	-	Positive
+	-	-	Positive
-	(+)	+	Positive
(+)	-	+	Positive
(+)	+	-	Positive
+	(+)	-	Positive
-	+	+	Positive
+	-	+	Positive
+	+	-	Positive
(+)	(+)	+	Positive
(+)	+	+	Positive
+	(+)	+	Positive
+	+	+	Positive

Supplementary Table 4. Overall ReproTracker classification. Possible scenarios of teratogenicity prediction outcomes when combining the trilineage differentiation assay calls (cardiomyocytes, hepatocytes, neural rosettes) for final compound prediction.

Cardiomyocytes	Hepatocytes	Neural rosettes	Teratogenicity prediction
Negative	Negative	Negative	Negative
Negative	Negative	Equivocal	Equivocal
Negative	Equivocal	Negative	Equivocal
Equivocal	Negative	Negative	Equivocal
Negative	Equivocal	Equivocal	Equivocal
Equivocal	Negative	Equivocal	Equivocal
Equivocal	Equivocal	Negative	Equivocal
Equivocal	Equivocal	Equivocal	Equivocal
Negative	Negative	Positive	Positive
Negative	Positive	Negative	Positive
Positive	Negative	Negative	Positive
Negative	Equivocal	Positive	Positive
Negative	Positive	Equivocal	Positive
Equivocal	Negative	Positive	Positive
Equivocal	Positive	Negative	Positive
Positive	Negative	Equivocal	Positive
Positive	Equivocal	Negative	Positive
Negative	Positive	Positive	Positive
Positive	Negative	Positive	Positive
Positive	Positive	Negative	Positive
Equivocal	Equivocal	Positive	Positive
Equivocal	Positive	Equivocal	Positive
Positive	Equivocal	Equivocal	Positive
Equivocal	Positive	Positive	Positive
Positive	Equivocal	Positive	Positive
Positive	Positive	Equivocal	Positive
Positive	Positive	Positive	Positive

Supplementary Table 5. Comparison between 24-well plate and 96-well plate formats of the ReproTracker Assay.

	24WP				96WP			
Compound Name	CM	LVR	NRL	LOAEL (μM)	CM	LVR	NRL	LOAEL(μM)
**Hydroxyurea**	NT	T	T	< 3.9	NT	T	T	≤ 2.0
**Saccharin**	NT	NT	NT	N.A.	NT	NT	NT	N.A.
**Thalidomide**	T	T	NT	1.6	T	T	NT	≤ 0.4
**Cyclophosphamide**	NT	NT	T	33	NT	NT	T	37.5
**Fluconazole**	NT	NT	T	250	NT	NT	T	125
**5-Fluorouracil**	NT	NT	NT	N.A.	NT	NT	NT	N.A.
**Isotretinoin**	T	T	T	≤0.0041	T	T	T	<0.0625
**Phenytoin**	NT	T	T	≤ 62.5	NT	T	T	31.3
**Cetirizine**	NT	NT	NT	N.A.	NT	NT	NT	N.A.
**Saxagliptin**	NT	NT	NT	N.A.	NT	NT	NT	N.A.

Supplementary Table 6. Highest non-cytotoxic concentration tested per compound based on the results from the dose range finding experiments.

Compound Name	Highest non-cytotoxic concentration (µM)
Acitretin	3.9
Aspirin	1000
Bosentan	62.5
Busulfan	2
Carbamazepine	125
Cisplatin	0.0258
Clarithromycin	100
Cyclophosphamide	300
Cytarabine	0.1
Dabrafenib	0.1
Dasatinib	0.0005
Dexamethasone	15.6
Diltiazem	125
Fingolimod	3.9
Fluconazole	1000
5-Fluorouracil	1.3
Flusilazole	125
Hydroxyurea	31.3
Ibrutinib	1.7
Ibuprofen	500
Imatinib	25
Isotretinoin	1
Lenalidomide	1000
Methimazole	1000
Methanol	1000
Methotrexate	0.1
Methylmercury	0.31
Mirex	0.625
Pazopanib	2
Phenytoin	125
Pomalidomide	200
Ribavirin	15.6
Tacrolimus	3.9
Thalidomide	50
Topiramate	1000
Tretinoin (Retinoic acid)	5
Trimethadione	1000
Valproic acid	500
Vismodegib	62.5
Warfarin	1000
Amoxicillin	500
Cetirizine	250
Folic acid	1.95
Hydrochlorothiazide	1000
Metoclopramide	62.5
1,2-propylene glycol	1000
Saccharin	250
Saxagliptin	200
Sitagliptin	1000
Thiamine	1000
Vildagliptin	250
